# Association of Water Intake with Hand Grip Strength in Community-Dwelling Older Adults

**DOI:** 10.3390/nu13061756

**Published:** 2021-05-21

**Authors:** Hyeonmok Kim, Sun Hee Beom, Tae Ho Kim, Beom-Jun Kim

**Affiliations:** 1 Division of Endocrinology and Metabolism, Department of Internal Medicine, Seoul Medical Center, Seoul 02053, Korea; hmkim0405@gmail.com (H.K.); whitesh11@hanmail.net (S.H.B.); drtaeho76@gmail.com (T.H.K.); 2 Division of Endocrinology and Metabolism, Department of Internal Medicine, Asan Medical Center, University of Ulsan College of Medicine, Seoul 05505, Korea

**Keywords:** water intake, hand grip strength, sarcopenia, muscle strength

## Abstract

Although recent clinical studies have suggested that water intake enhances muscle mass, its impact on muscle strength remain unclear, especially in older adults. This cross-sectional, population-based study using a representative sample of Koreans investigated the relationship of water intake with hand grip strength (HGS) in 4443 older adults, including 2090 men aged ≥50 years and 2253 postmenopausal women. A digital grip strength dynamometer was used for HGS assessment. Low muscle strength was defined by the Korean-specific HGS cut-off value and adequate water intake was defined according to the Korean dietary reference intakes. In an unadjusted model, water intake was significantly higher in men and women without than with low muscle strength (both *p* < 0.001), but this difference disappeared after adjustment for confounding variables in both men (*p* = 0.050) and women (*p* = 0.245). Similarly, the correlation between water intake and HGS, the difference in HGS depending on adequate water intake status, and the risk of low muscle strength depending on water intake quartile were significant only in the unadjusted model. These data indicate that factors such as age, body size, and resistance exercise contribute to improvements in HGS in older adults, whereas water intake may not.

## 1. Introduction

The increased life span of individuals has led to increases in the incidence of sarcopenia, which has become a major healthcare problem, especially in older adults [1,2]. Muscle mass and muscle quality are the two main factors determining muscle strength, with muscle quality deteriorating faster than muscle mass with age [3]. Because muscle mass contributes only about 5% to muscle strength, evaluating muscle quality is very important in assessing and treating sarcopenia [4]. Hand grip strength (HGS), a fast and simple measurement of maximum voluntary force, is a reliable indicator of muscle quality [5,6], and the European guideline has recommended HGS as a key index for low muscle strength in the sarcopenia diagnosis [7].

Adequate intake of certain nutrients is essential to maintain body health and is considered a modifiable factor for sarcopenia prevention. Water is regarded as the most important nutrient associated with sarcopenia, both because approximately 76% of muscle mass consists of water [8] and because water is involved in almost all biological and chemical reactions in muscle [9]. Studies in dehydrated mice have shown that skin and muscle are the first major organs to lose water, thereby protecting other vital organs including the brain and liver [10,11]. Furthermore, dehydration, or the reduction in total body water, was found to have detrimental effects on muscle mass and endurance exercise performance, with these detrimental effects overcome through oral rehydration [12,13,14]. Although several epidemiologic studies have evaluated the role of water intake as a modifiable risk factor for sarcopenia [15,16,17], those studies have generally evaluated muscle mass, a factor contributing only partially to muscle strength. To clarify the possible impact of water intake on muscle strength, the current study investigated the relationship between water intake and HGS in a representative sample of older Koreans.

## 2. Materials and Methods

### 2.1. Study Participants

This study was cross-sectionally designed using data acquired during the Sixth Korean National Health and Nutrition Examination Survey (KNHANES VI), which was conducted from 2014 to 2015 [18]. To select a representative sample of the civilian, non-institutionalized Korean population, this survey adopted a clustered, multistage, stratified probability sampling method. The KNHANES VI is composed of three parts: a health examination survey, a nutrition survey, and a health interview survey. Informed consent was obtained from all participants in KNHANES VI. The database is open to the public at the website (http://knhanes.cdc.go.kr/knhanes/eng accessed on 25 April 2021).

The subjects included in this study consisted of men aged ≥50 years and postmenopausal women, all of whom were taken from the KNHANES VI database. Subjects with neoplastic disease, myocardial infarction, stroke sequel, renal failure (estimated glomerular filtration (eGFR) <60 mL/min/17.3 m^2^), or high serum liver enzyme level (alanine aminotransferase or aspartate aminotransferase >100 IU/L) were excluded.

### 2.2. Measurements of HGS

A digital grip strength dynamometer measuring forces between 5.0 and 100.0 kg in units of 0.1 kg was used to assess HGS (T.K.K. 5401, Takei Scientific Instruments Co., Ltd., Tokyo, Japan). This dynamometer is based on an electro-mechanical system with a digital display and a complacent, rectified, and adjustable hand shape [19]. The calibration of instruments was performed by the gradual application of factual loads. During the measurement, participants were instructed to look forward with the elbow fully extended and to stand upright with their feet hip-width apart. In a comfortable, neutral position (not extended or flexed), participants held the dynamometer with the testing hand. Participants always started with the dominant hand and performed three trials with each hand alternately. Participants were asked to squeeze the grip continuously with full force for at least 3 s. The time between each trial was about 60 s. The maximally measured grip strength of the dominant hand was recorded [20]. HGS was evaluated using the Korean-specific cut-off points of 28.6 kg in men and 16.4 kg in women, according to the population-based study [20].

### 2.3. Assessment of Water Intake and Definition of Adequate Water Intake

Levels of water intake were determined from the nutrition survey, with water intake of subjects in KNHANES VI based on measurements of 1 cup (200 mL). Adequate water intake under standard conditions was defined according to the Korean dietary reference intakes >1000 mL per day in men and >900 mL per day in women [21].

### 2.4. Measurements of Dietary, Laboratory and Clinical Parameters

All participants in KHANES VI completed a health interview and a health examination survey, which included the subjects: educational level, level of house income, smoking status, alcohol status, resistance exercise, dietary intake and anthropometric measurements. While the participants were dressed in light clothing without shoes, anthropometric factors such as weight (kg) and height (cm) were evaluated by standardized protocols. Body mass index (BMI; kg/m^2^) was generated from each subject’s weight and height. Smoking was categorized as never, former, or current. Resistance exercise, such as horizontal bars, dumbbell exercise, sit-ups, and push-ups, was also classified into three levels: none, intermittent (1 to 3 days per week), or regular (≥4 days per week). A standard drink was defined as a single glass of liquor, wine, beer, or the Korean traditional drink so-ju (Korean distilled liquor). At least seven drinks at one time in men or at least five drinks at one time in women, each more than twice per week was defined as heavy alcohol drinking.

A 24 h dietary recall questionnaire administered by a trained dietitian was used to evaluate the nutritional status, including protein intake (g/day) and total energy intake (kcal/day), which were based on the Food Composition Table [22].

After an overnight fast for ≥8 h, blood samples were collected from all participants. These blood samples were transported to the Central Testing Institute in Seoul, Korea, with immediate refrigeration, and analyzed within 24 h of receipt. Serum creatinine level was determined colorimetrically using a Hitachi Automatic Analyzer 7600 (Tokyo, Japan; reference range: 44–80 μmol/L for women and 53–106 μmol/L for men). eGFR was estimated by the Modification of Diet in Renal Disease Study formula [23]:

eGFR (mL/min/1.73 m^2^) = [186 × serum creatinine (mg/dL)^−1.154^] × age^−0.203^ (× 0.742 for women). (1)

### 2.5. Statistical Analysis

Categorical variables are expressed as number (percentage) and compared by chi-square tests, whereas continuous variables are presented as mean ± standard error (SE) and compared by unpaired *t* tests. Univariate- and multivariate-adjusted, least-square means with 95% confidence intervals (CI) of water intake in subjects with and without low muscle strength were estimated and compared by analysis of variance (ANOVA) and covariance (ANCOVA), respectively. The potential confounders were selected on the basis of their clinical applicability. Specifically, age, BMI, smoking status, heavy alcohol drinking, resistance exercise, total energy intake, and protein intake were included in multivariate-adjusted model. The variance inflation factor and tolerance were below 10 and above 0.1, respectively, for all explanatory variables and thus we could confirm that the rule for multicollinearity among variables was not violated in the multivariate-adjusted analyses. To test the hypothesis that water intake could be independently related to HGS, linear regression analyses were performed using an entry method, with water intake as the independent variable and HGS as the dependent variable. Participants were classified into groups according to their levels of water intake, followed by logistic regression analyses to generate odds ratio (95% CI), before and after adjustment for confounders. The univariate- and multivariate-adjusted least-square means with 95% CIs of HGS in subjects with and without adequate water intake were also estimated and compared by ANOVA and ANCOVA, respectively. Sample weighting using the Complex Samples Plan (CSPLAN) in high SPSS version (IBM, Inc., Armonk, NY, USA) was applied in all analyses. *p* < 0.05 was considered statistically significant.

## 3. Results

### 3.1. Clinical Characteristics of the Study Participants

Data on HGS and water intake were available for 2246 men aged ≥50 years and 2506 postmenopausal women who participated in KNHANES VI. Of these 4752 subjects, 309 had neoplastic disease, myocardial infarction, stroke sequel, renal failure, or high serum liver enzyme level and were therefore excluded. Data from the remaining 4443 subjects, including 2090 (47.0%) men and 2353 (53.0%) postmenopausal women, were analyzed. The baseline characteristics of these subjects are presented in Table 1. The mean ages of the men and women subjects were 64.79 years (range: 50–80 years) and 64.12 years (range: 39–80 years), respectively. Both men and women with low muscle strength were shorter in height; lower in weight; and had lower educational level, household income, participation in resistance exercise, and total energy and protein intake (*p* < 0.001 each) than subjects without low muscle strength. BMI was lower in men (*p* < 0.001), but not in women (*p* = 0.352), with than without low muscle strength. By contrast, heavy alcohol drinking was more frequent in women (*p* < 0.001) but not in men (*p* = 0.771) with than without low muscle strength. Age was greater in men and women with than without low muscle strength. Current smoking was less frequent in men, but more frequent in women, with than without low muscle strength.

### 3.2. Association between Water Intake and Low Muscle Strength

**Figure 1:**
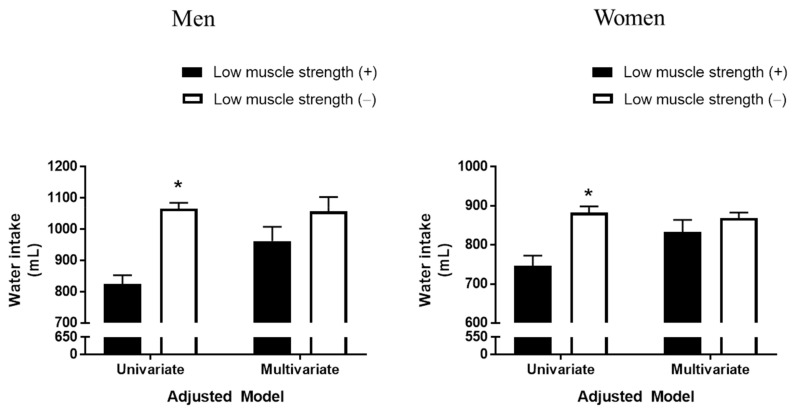
Differences in water intake in men and women with and without low muscle strength before and after adjustment for confounders. Results are expressed as estimated means with 95% confidence intervals based on analysis of covariance. Confounding factors included age, body mass index, smoking status, resistance exercise, heavy alcohol drinking, protein intake, and total energy intake. The cutoffs for hand grip strength defining low muscle strength were 28.6 kg in men and 16.4 kg in women. * Statistically significantly different from subjects with low muscle strength.

Before adjustment for confounding variables, such as age, BMI, smoking status, alcohol status, regular exercise status, protein intake, and total energy intake, water intake was significantly lower in both men (824.43 mL vs. 1065.86 mL; *p* < 0.001) and women (746.35 mL vs. 882.36 mL; *p* < 0.001) with than without low muscle strength (Figure 1). After adjustment for these confounding variables, however, water intake was similar in both men (*p* = 0.050) and women (*p* = 0.245) with and without low muscle strength.

### 3.3. Correlation of HGS with Clinical Parameters

The covariates that were independently related to HGS was assessed by linear regression analyses (Table 2). Before adjustment, in both men and women, age was inversely associated with HGS (*p* < 0.001), whereas BMI, resistance exercise, and total energy intake were positively correlated with HGS (*p* < 0.001 to *p* = 0.048). Water intake also has a positive association with HGS in both genders (*p* < 0.001). Interestingly, after adjustment for confounders, water intake was no longer significantly associated with HGS in both men (*p* = 0.379) and women (*p* = 0.233). However, several other variables, including age, BMI, and resistance exercise, continued to have significant associations with HGS in both genders (*p* < 0.001 to *p* = 0.006).

### 3.4. Risk of Low Muscle Strength According to Water Intake Amount

**Figure 2:**
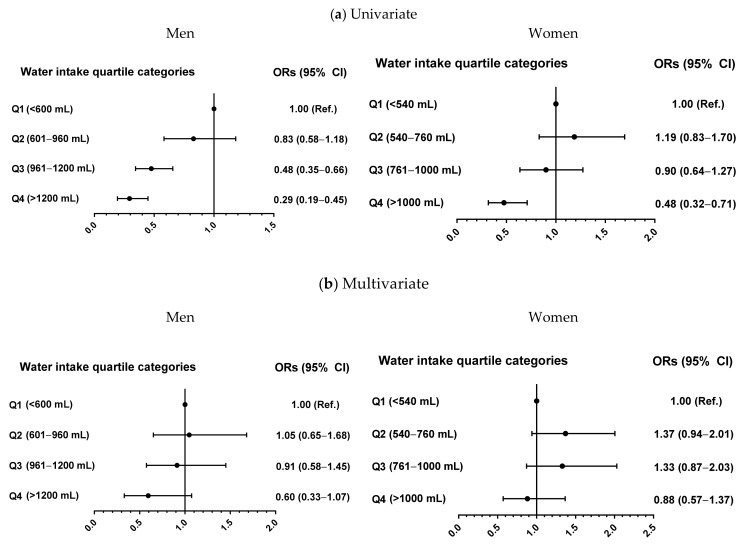
Odds ratios (95% confidence intervals) for low muscle strength depending on water intake quartiles before (**a**) and after (**b**) adjustment for confounding variables in men and women by logistic regression analyses. Confounding factors included age, body mass index, resistance exercise, smoking status, heavy alcohol drinking, protein intake, and total energy intake. The cutoffs for hand grip strength defining low muscle strength were 28.6 kg in men and 16.4 kg in women. Abbreviations: ORs, odds ratios.

To further evaluate the relationship of water intake with low muscle strength, subjects were classified into four groups depending on their amount of water intake, and logistic regression analysis was performed (Figure 2). In univariate-adjusted models, the odds ratios for the risk of low muscle strength in men and women in quartile 4 were 0.29-fold and 0.48-fold lower, respectively, than in men and women in quartile 1. The odds ratios were no longer statistically significant in both genders after adjustment for confounder, including age, BMI, regular exercise status, smoking status, heavy alcohol drinking, protein intake, and total energy intake.

### 3.5. Effects of Adequate Water Intake on HGS and Risk of Low Muscle Strength

**Figure 3:**
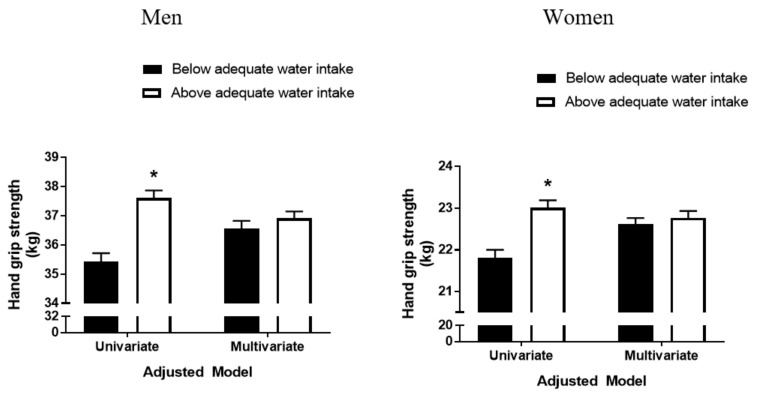
Differences in hand grip strength in men and women with below and above adequate water intake before and after adjustment for confounders. Results are expressed as estimated means with 95% confidence intervals based on analysis of covariance. Confounding factors included age, body mass index, resistance exercise, smoking status, heavy alcohol drinking, protein intake, and total energy intake. The cutoffs defining adequate water intake were >1000 mL in men and >900 mL in women. * Statistically significantly different from subjects with below adequate water intake.

Adequate water intake under standard conditions in Koreans has been defined as >1000 mL per day in men and >900 mL per day in women [21]. Men and women were each divided into those with above and below adequate water intake groups and HGS was compared in these groups (Figure 3). Before adjustment for confounding variables, HGS was significantly lower in men (35.44 kg vs. 37.61 kg; *p* < 0.001) and women (21.81 kg vs. 23.01 kg; *p* < 0.001) with below than above adequate water intake. After adjustment for confounding variables, differences in HGS according to the amount of water intake disappeared in both men (*p* = 0.280) and women (*p* = 0.477).

**Figure 4:**
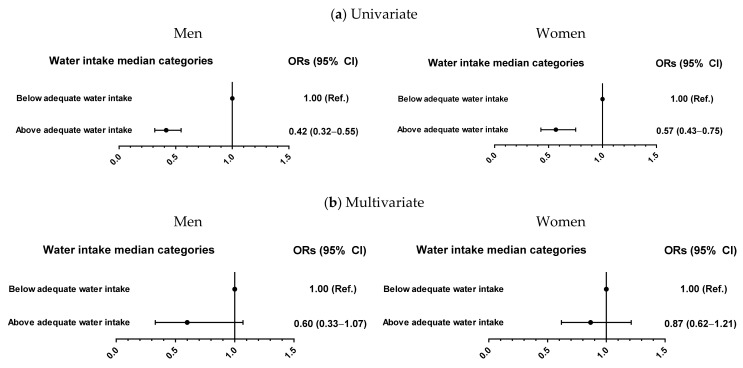
Odds ratio (95% confidence interval) for low muscle strength according to median water intake (**a**) before and (**b**) after adjustment for confounding variables in men and women by logistic regression analyses. Confounding variables included age, body mass index, smoking status, heavy alcohol drinking, resistance exercise, total energy intake, and protein intake. The cutoffs defining adequate water intake were >1000 mL in men and >900 mL in women. The cutoffs for hand grip strength defining low muscle strength were 28.6 kg in men and 16.4 kg in women. Abbreviations: ORs, odds ratios.

Logistic regression analyses were used to generate odds ratio comparing the likelihood of low muscle strength in subjects with below compared with above adequate water intake. This analysis also showed that the statistical significance of odds ratios in both men and women disappeared after adjustment for confounding factors (Figure 4).

## 4. Discussion

Univariate analysis of a representative sample of older Koreans showed that water intake differed significantly in subjects with and without low muscle strength, with the statistical significance disappearing after adjustment for potential confounding variables. Similarly, the correlation between water intake and HGS, the difference in HGS between subjects with and without adequate water intake, and the risk of low muscle strength according to water intake quartile were significant only on univariate analyses. These findings suggest water intake may not be associated with improved HGS in older adults; rather, factors such as age, resistance exercise, and BMI contribute to greater HGS in these subjects.

Despite having high muscle mass, older obese individuals usually have a higher incidence of falls and a lower physical performance than non-obese older adults [24]. Furthermore, cross-sectional studies in older adults and across the adult lifespan have shown that the estimated rate of muscle mass loss is about 0.4% to 2.6% per year, whereas the estimated rate of muscle strength loss is higher, ranging from 1% to 3% per year [25,26]. These findings suggest that factors other than muscle mass, such as muscle aerobic capacity, intramuscular adipose tissue, and motor units and neuromuscular activation, have greater influence on muscle strength and the risk of falling. In the current study, we used very simple and cost effectiveness method, HGS which provide swift estimates of muscle quality. Actually, previous studies have shown that HGS but not muscle mass is related to fall, mortality, and hospitalization risk. These results suggest a shift of focus on increasing muscle quality and improving physical function rather than only increasing muscle mass [27,28]. By using HGS, we expected to be able to assess the association of water intake more clearly with muscle strength in older adults.

When interpreting our results, the shortcoming of water intake as an indicator of human hydration status should be considered. The clinical importance of water should be evaluated based on hydration status of individuals. Although several indicators, such as urine color, changes of daily weight and urine output, skin turgor, dry mouth, and blood levels of sodium, chloride, urea nitrogen, creatinine, and osmolality, have been suggested [29], these factors may not be easily assessed in large epidemiologic studies. Furthermore, there is no generalized consensus of gold standard methods for hydration status assessment. As the next best way, water intake has been frequently used on the topic related to the roles of water in human health [29]. However, hydration level is affected by various factors such as ambient environment, sweat rates, physical activity, energy expenditure, and so on, and thus water intake alone could not provide enough information on actual hydration status in human body. Consequently, there is a possibility that our results could be biased by the methodologic limitation of water intake.

Although several studies have reported that water intake has associations with sarcopenia and low physical performance in older adults, our study found no significant correlation of water intake with HGS. These results may have been due to the effects of several confounding factors, such as age, BMI, and resistance exercise. Age is regarded as the most important factor affecting HGS in healthy subjects, because HGS declines with age [30]. The present study also found the inverse relationship of age with HGS in both men and women, regardless of adjustment for confounding factors. This study also showed that BMI and resistance exercise, two other important determinants of HGS [31,32], are independent factors positively correlated with HGS. Several studies investigating the effect of hydration on muscle strength were unable to completely rule out the effect of weight gain due to water intake. Thus, adjustments for age, BMI, and resistance exercise might be strong enough to offset the effect of water intake on muscle metabolism. Although increased water intake can enhance muscle strength, the results of the present study suggest that water intake might not be the critical determinant of muscle strength in older adults after considering confounding variables, such as age, BMI, and resistance exercise.

The major strength of this study was that it was a population-based study using systematically collected and representative national data, enhancing the generalizability and statistical reliability of the results. This study utilized the specific HGS cut-off values for Korean to define low muscle strength as recommended by the sarcopenia guideline [33,34] because these cut-offs can depend on race, body size, living habits, and cultural backgrounds. However, this study also had several limitations. First, its cross-sectional design does not permit conclusions to be drawn regarding causality. Furthermore, all the participants were of the same ethnicity so the interpretation is restricted to the population of the Republic of Korea. Second, daily water and protein intake data used in our study were based on a 24 h dietary recall method. Despite the usefulness of this method, especially in a large study, the use of self-reported questionnaires could result in faulty recall or misinterpretation of absolute water and protein intake amounts. Lastly, although this study included as many covariates as possible, the observed results could have been attributable to uncontrolled medications or comorbidities that influence muscle metabolism.

In conclusion, data gathered from a nationally representative sample indicate that water intake has a significant association with HGS only on univariate analysis, with the statistical significance disappearing after adjustment for potential confounding variables, including age, BMI, and resistance exercise. These findings suggest that water intake might not be the critical determinant of muscle strength in older adults.

## Figures and Tables

**Table 1:** Baseline characteristics of the study participants.

Variables	Men (*n* = 2090)			Women (*n* = 2353)		
	Low Muscle Strength			Low Muscle Strength		
	(+; *n* = 379)	(−; *n* = 1711)	*p* Value	(+; *n* = 343)	(−; *n* = 2010)	*p* Value
Age, y	**73.14 ± 0.36**	**62.94 ± 0.20**	**<0.001**	**71.85 ± 0.42**	**62.80 ± 0.20**	**<0.001**
Height, cm	**162.68 ± 0.29**	**167.51 ± 0.14**	**<0.001**	**149.50 ± 0.33**	**154.55 ± 0.13**	**<0.001**
Weight, kg	**60.15 ± 0.47**	**68.03 ± 0.23**	**<0.001**	**53.88 ± 0.46**	**57.91 ± 0.19**	**<0.001**
Body mass index, kg/m^2^	**22.71 ± 0.16**	**24.21 ± 0.07**	**<0.001**	24.06 ± 0.18	24.24 ± 0.07	0.352
Educational level, %			**<0.001**			**<0.001**
Less than high school	**70.3**	**44.5**		**87.3**	**61.0**	
High school or advanced	**29.7**	**55.5**		**12.7**	**39.0**	
Household income quartile, %			**<0.001**			**<0.001**
1 (lowest)	**51.3**	**20.3**		**55.8**	**27.3**	
2	**25.5**	**28.2**		**22.5**	**26.7**	
3	**12.8**	**24.6**		**13.7**	**21.9**	
4	**10.4**	**26.9**		**8.0**	**24.1**	
Smoking status, %			**<0.001**			**0.001**
Never	**28.7**	**21.7**		**92.2**	**94.6**	
Former	**53.8**	**49.4**		**5.0**	**2.8**	
Current	**17.5**	**28.9**		**2.9**	**2.6**	
Heavy alcohol drinking, %			0.771			**<0.001**
Yes	16.9	16.1		**6.4**	**3.2**	
No	83.1	83.9		**93.6**	**96.8**	
Resistance exercise, %			**<0.001**			**<0.001**
No	**80.8**	**67.6**		**95.9**	**84.1**	
Intermittent	**5.7**	**13.5**		**2.6**	**8.5**	
Regular	**13.6**	**18.9**		**1.5**	**7.4**	
Total energy, kcal/d	**1811.65 ± 37.92**	**2264.79 ± 23.31**	**<0.001**	**1447.22 ± 34.88**	**1692.41 ± 15.64**	**<0.001**
Total proteins, g/d	**55.79 ± 1.48**	**77.43 ± 1.55**	**<0.001**	**45.32 ± 1.43**	**56.21 ± 0.66**	**<0.001**

Data are expressed as percentage for categorical variables and as mean ± standard error for continuous variables. Bold fonts indicate statistically significance differences. The cutoffs for hand grip strength defining low muscle strength were 28.6 kg in men and 16.4 kg in women.

**Table 2:** Covariates independently associated with hand grip strength by linear regression analyses.

	Unadjusted for Covariates			Adjusted for Covariates		
Independent Variables	ß	SE	*p* Value	ß	SE	*p* Value
Men						
Age	**−0.453**	**0.016**	**<0.001**	**−0.398**	**0.017**	**<0.001**
Body mass index	**0.591**	**0.063**	**<0.001**	**0.363**	**0.059**	**<0.001**
Smoking status	−0.002	0.001	0.112	**−0.444**	**0.190**	**0.020**
Heavy alcohol drinking	−0.002	0.001	0.113	0.084	0.178	0.638
Resistance exercise	**0.636**	**0.249**	**0.011**	**0.642**	**0.234**	**0.006**
Total energy intake	**0.002**	**0.001**	**<0.001**	**0.001**	**0.000**	**0.016**
Protein intake	0.022	0.012	0.054	0.000	0.005	0.964
Water intake	**0.002**	**0.000**	**<0.001**	0.000	0.000	0.379
Women						
Age	**−0.266**	**0.012**	**<0.001**	**−0.257**	**0.012**	**<0.001**
Body mass index	**0.080**	**0.040**	**0.048**	**0.130**	**0.037**	**<0.001**
Smoking status	**−0.465**	**0.138**	**0.001**	−0.248	0.151	0.102
Heavy alcohol intake	**−0.322**	**0.152**	**0.035**	0.056	0.168	0.741
Resistance exercise	**1.076**	**0.172**	**<0.001**	**0.539**	**0.165**	**0.001**
Total energy intake	**0.001**	**0.000**	**<0.001**	0.000	0.000	0.702
Protein intake	**0.024**	**0.006**	**<0.001**	0.001	0.009	0.944
Water intake	**0.001**	**0.000**	**<0.001**	0.000	0.000	0.233

Bold fonts indicate statistically significance differences.

## Data Availability

The database for this survey is publicly available at the KNHANES website (http://knhanes.cdc.go.kr/knhanes/eng accessed on 25 April 2021; available in English).
